# Genetic improvement of robusta coffee (*Coffea canephora*) in the 20th and 21st centuries: prospects for increasing future breeding effectiveness and impact

**DOI:** 10.3389/fpls.2025.1719980

**Published:** 2026-01-15

**Authors:** Robert Kawuki, Abraham Akpertey, Santos Barrera, Jorge C. Berny Mier y Teran, Jeena Devasia, Kraig Kraft, Miftahur Rizqi Akbar, B.R. Shivalingu, Godfrey Sseremba, Alexsandro Teixeira, Rodrigo Barros Rocha, Phan Viet Ha, Ari Wibowo, Tania Humphrey

**Affiliations:** 1 World Coffee Research, Portland, OR, United States; 2 Cocoa Research Institute of Ghana, New Tafo-Akim, Ghana; 3 Central Coffee Research Institute, Coffee Research Station, Karnataka, India; 4 Indonesian Coffee and Cocoa Research Institute (ICCRI), East Java, Indonesia; 5 National Agricultural Research Organisation (NARO), Entebbe, Uganda; 6 Embrapa Cafe, Brazilian Agricultural Research Corporation, Brasilia, Brazil; 7 Institute of Research, Technical Assistance and Rural Extension of Espirito Santo (INCAPER), Vitoria, Brazil; 8 Western Highlands Agriculture and Forestry Science Institute (WASI), Buon Ma Thuot, Vietnam

**Keywords:** *Coffea canephora*, breeding programmes, genetic gains, germplasm, origins, selection, target product profiles, varieties

## Abstract

Robusta coffee (*Coffea canephora*), with annual global production ranging from 70 to 76 million bags, has a long history of breeding and crop improvement worldwide. However, breeding efforts to address issues such as subpar farmer profitability and low production per robusta tree still rely on time-consuming approaches that have not changed in decades, making it difficult to respond to evolving needs and market demands. Operationally, the most urgent challenges in robusta breeding are the >20-year variety development time, the limited amount of germplasm with elite traits and heritable genetic variation, and the lack of transdisciplinary collaboration. In this review, we lay the groundwork to address these challenges. We examine pioneering robusta breeding programs in Africa (Uganda, Ivory Coast, Cameroon and Ghana), Asia (Indonesia, India, and Vietnam), and Latin America (Brazil), focusing on trait priorities, the breeding methods employed, the types of varieties developed and disseminated, and the genetic knowledge generated, as well as notable missteps and lessons learned. We then highlight and discuss opportunities for improving robusta breeding efficiency and impact with a specific focus on i) the use of clear target product profiles as guidelines for operationalizing demand-led robusta breeding; and ii) strategies to drive and deploy genetic gains in farmer field. Further we highlight entry points that maximize the impact of integrating modern breeding tools (such as genomics-assisted selection and instrument-based phenotyping) to enhance breeding operations. Our view is based on the understanding that effective breeding integrates processes, products, and human skill at a reasonable cost and within a reasonable timeframe to overcome current and anticipated challenges, particularly those brought on by climate change. Finally, we advocate for multilateral collaborations among industry, academia, and governments, as a powerful approach to bolster the development of the much-needed innovations for farmers. We hope that this retrospective review, from which we derive interventions, will improve the effectiveness and impact of robusta breeding at scale.

## 1 Introduction

Coffee is one of the most widely consumed beverages in the world, with an estimated 400 billion cups consumed every year (ICO, 2025). In 2023 alone, global coffee production was worth USD 23 billion and international trade was worth USD 26 billion; the entire industry generates more than USD 200 billion a year (FAO, 2025). Of the 133 known *Coffea* species, only two species, arabica coffee (*Coffea arabica*) and robusta coffee (*Coffea canephora*, robusta hereafter), are widely cultivated and consumed, with arabica having a greater market share than robusta (Eskes and Leroy, 2004; FAO, 2025).

The global production of robusta now accounts for 40% of all coffee production, up from 25% in the early 1990s. This increase has had three notable effects on the robusta production-processing-consumption value-chain continuum over the last 40 years: counteracting poverty, improving gender and social inclusion, and contributing towards mitigating the negative effects of climate change (Mbowa et al., 2017; Hung Anh et al., 2019; Ferrão et al., 2024; Kashaija et al., 2025). India is a shining example of this success. For example, coffee cultivation during the 1950s consisted of about 72% arabica and 28% robusta. However, several profit-based factors (such as new markets opportunities for optimally-produced specialty robusta) have triggered the gradual shift from arabica toward robusta, which now stands at 48% of coffee-growing acreage in India (ICO, 2024). The increased adoption of robusta in the Kerala and Karnataka coffee growing region is credited as one of the interventions that lifted many out of deep poverty in India. Similar societal shifts have also been noted in northeastern Brazil, particularly in the robusta-growing regions of Espírito Santo, Rondônia and Bahia.

On the research end, robusta breeding faces significant ongoing challenges, and the decades-old breeding approaches currently used are inadequate to respond efficiently to emerging market needs and other evolving challenges. These challenges include decreasing profitability for farmers (Kiyingi and Gwali, 2012; Martinez, 2023; Ngure and Watanabe, 2024), the over 20-year development time for new varieties to be approved for commercial production (Eskes and Leroy, 2004) and the unrelenting negative effects of climate change on production and productivity (Ogundeji et al., 2019; Kath et al., 2020; Ngure and Watanabe, 2024). The negative impacts of these challenges vary markedly among countries and continents and thus urgently require lasting solutions. For example, prolonged drought stress and high temperatures in Uganda increase robusta’s vulnerability to both black coffee twig borers (*Xylosandrus compactus*) and coffee wilt disease (Karungi et al., 2021). Relatedly, higher soil temperatures and drought stress in Asia and Brazil increase robusta’s vulnerability to root-knot nematodes (Marengo, 2008; Hoang et al., 2021). Climate change is likely to reorder pest and disease threats to robusta and warrants concerted research effort to mitigate its negative effects.

The development of practical and lasting solutions to meet these challenges requires a thorough examination of the current state of knowledge to help inform future interventions. This analysis should integrate empirical studies and the lessons—both good and bad—learned from practical and repeated deployment in the field, with the ultimate goal of increasing robusta production and profitability. This analysis should be conducted separately for different parts of the world, both where coffee originated (in Africa) and where it has been introduced (i.e., in Asia and Latin America). Here, we undertake a retrospective analysis of robusta genetic improvement from early 1900s to the current day as a foundation to inform strategies aimed at breeding robusta for the future.

To ensure that future breeding investments yield high returns, Brennan and Martin (2007) highlighted several questions that must be answered: Which traits should be targeted? What trait information can be used early in the selection process? Which traits are associated with lower costs of introgression, evaluation and selection? Which technologies and/or techniques may be deployed most effectively? And what infrastructure and operational changes are needed to adopt new technology? To respond to some of these questions, we begin by documenting the current state of knowledge in robusta breeding as outlined hereafter.

The roots of robusta—a coffee species plant visually recognizable by its uniquely large blossoms and wide, spreading canopy—trace back hundreds of years to the understory forests in tropical areas of central and western sub-Saharan Africa, a region with expansive geographic distribution from Guinea in West Africa, to Uganda, in East Africa and then to Angola, on the western coast of southern Africa (Gomez et al., 2009). Throughout history, robusta has been grown in numerous forms and ecotypes, and in regions beyond its birthplace. Currently, robusta is grown in more than 20 countries, which are characterized by warm climates and/or high humidity, and has dramatically evolved into a major market force.

Robusta was exported from Africa in the late 1800s (Herrera and Lambot, 2017). Before then, indigenous people likely cultivated and exploited the species in different locations, as evidenced by informal selection of individual trees from wild populations in the Ugandan forests (Thomas, 1935). Often ripe berries could be picked from trees and not used as a beverage, but for chewing after being boiled and dried; trees providing outstanding berries were used as source of seed for establishing new plantations (Thomas, 1935). This informal selection, also done at different robusta birthplaces, inadvertently exploited the outcrossing nature of robusta, thus helping wild individuals adapt to new environments and meet local needs and markets. It is thus possible that robusta exported from Africa consisted of wild and semi-wild individuals, which were later subjected to additional selection.

From its original location, robusta was exported to various destinations in Asia and Latin America for commercial coffee production and for the initial systematic genetic improvement of the species. For instance, robusta was introduced in Asia after a coffee leaf rust epidemic caused by the fungus *Hemileia vastatrix* devastated local arabica production. The international exchange and movement of robusta germplasm stimulated the start of formal robusta breeding efforts (**Table 1**). Pioneer robusta breeding programs in Africa, Asia, and Latin America (mostly Brazil), played crucial roles in long-distance robusta germplasm exchange, expanding production acreage, generation of scientific knowledge, and development of genetically-adapted clones and populations, all done within limits of available germplasm, technology and knowledge at the time (Thomas, 1935; Ferwerda, 1948; Leroy et al., 1993; Eskes and Leroy, 2004; Leroy et at., 2006; Leroy et al., 2011).

**Table 1:** The exchange of robusta genetic resources that initiated breeding across the globe.

Origin	Destination	Year	Inherent traits being sought	References
Democratic Republic of Congo (DRC)	Indonesia	1901	Coffee leaf rust (CLR) resistance, vigor, growth traits, productivity and bean size	(Herrera and Lambot, 2017; Ferwerda, 1948)
Indonesia	Uganda	1910	Growth traits, early fruiting, and bean size	(Thomas, 1935)
DRC, Indonesia	Vietnam	1900s	Growth and productivity traits	(Phan, 2017)
Congo, Indonesia	Brazil	1912	CLR resistance, drought tolerance	(Herrera and Lambot, 2017)
Indonesia	India	1916	Growth and productivity traits	(Herrera and Lambot, 2017)
Uganda	Indonesia	1916	Growth and productivity traits	(Ferwerda, 1948)
DRC	Uganda	1940 to 1944	Plant architecture (i.e. erect) and quality traits	(Davis et al., 2023 and references therein)
Guinea, Cameroon, and the DRC	Ivory Coast	1930 to 1960s	Agronomic and resilience traits	(Herrera and Lambot, 2017)

Robusta genetic resources used to initiate breeding across the globe are based on a few founders, most of which originated from central and/or eastern Africa region. Thus, sites of origin retain substantial genetic variation not used by breeding programs.

Following the global movement of germplasm, the genetic diversity of robusta was extensively studied. Two primary genetic groups, namely “Congolese” and “Guinean”, have been documented, based on their geographical origin and natural distribution in Africa (Akpertey et al., 2021; Kiwuka et al., 2021). Robusta trees from the Guinean group are characterized by narrow internodes, short stature, high caffeine content, low bean weight, drought resistance, and early harvesting, while those from the Congolese group exhibit rust resistance, intermediate caffeine content, high bean weight, susceptibility to drought, large internodes, low secondary branching, tall stature, and late harvesting (Leroy et al., 1993; Cilas et al., 2006).

The “conilon” group, predominantly grown in Brazil, defines another genetic group presumed to have resulted from hybridisation between Congolese and Guinean trees, as individuals within the conilon genetic group display phenotypes that are intermediate between its two presumed parental groups (Gomez et al., 2009; Silva et al., 2025). Current robusta cultivars (selected by breeding programs and/or by farmers), are derived from these three genetic groups, and thus shape the pool of robusta genetic diversity that is currently available.

In this review, we first summarize the breeding steps taken and milestones attained by pioneering robusta breeding programs in Africa (Uganda, Ivory Coast, Cameroon and Ghana), Asia (Indonesia, India, and Vietnam), and Latin America (Brazil). We focus on key features of these programs, including the priority traits of emphasis during selection, the types of varieties produced, the genetic knowledge gained, and the major lessons learned. Following this retrospective analysis, we discuss opportunities for enhancing robusta breeding efficiency, with a specific focus on the inclusion of target product profiles to guide and support demand-led breeding as well as strategies to drive and deploy genetic gains in farmer fields, based on the understanding that effective breeding integrates processes, products, and human skill at reasonable cost and time.

## 2 Pioneer robusta breeding programmes and strategies

Plant breeding has been an ever-evolving discipline since domestication, when our ancestors’ commenced selection for desirable traits in plants for food, medicine, fiber and/or other uses (Fuller et al., 2014). This informal selection was later replaced with more systematic plant breeding that built on pioneering work of Gregor Mendel that was conducted in mid-1800s (McLysaght, 2022). As a result of both new understanding and technological breakthroughs, plant breeding techniques and strategies have significantly changed since the early 2000s (Collard and Mackill, 2008; Hamdan et al., 2022; Jeon et al., 2023), greatly impacting both crops and livestock value chains across the globe. In the case of robusta, formal breeding only commenced in the early 1900s following the global movement of germplasm (**Table 1**).

As for other crops, breeding methods in robusta are influenced by inherent biological characteristics. For instance, robusta is heterozygous and predominantly outcrosses (Eskes and Leroy, 2004) and major genetic groups of robusta exhibit unique economically important traits (Leroy et al., 1993; Gomez et al., 2009). These features emphasize the importance of cross pollination (intra and/or inter specific hybridizations) to create genetic variation for selection, which can consequently be exploited using varied breeding methods notably: phenotypic mass selection, recurrent selection, backcrossing, synthetic and/or hybrid variety development. In addition, robusta is diploid (2n = 2× = 22), with a small genome (710 megabases) compared to other coffee species (Denoeud et al., 2014). Based on these characteristics, we predict that targeted inter-specific hybridizations with other diploid coffee species should be possible, and that genome-derived technologies should be relatively less complex to develop and adopt. Indeed, the robusta genome has been sequenced (Denoeud et al., 2014), providing a foundation for genomic applications. Notably, unpollinated stigmas of robusta remain fertile for up to 10 days, while mature pollen is viable for only two days (Eskes and Leroy, 2004). Thus, anthesis synchronization, stigma receptibility and pollen viability will be essential considerations when deploying varieties in farmers field and/or in operationalizing polycross nurseries.

With these inherent biological characteristics that influence breeding methods in mind, we highlight pioneering robusta breeding programs in Asia (Indonesia, India and Vietnam), Africa (Uganda, Ghana and Ivory Coast), and Latin America (Brazil). Notable features of other operating private and public robusta breeding institutions are also provided. We aim to gain insight into trait priorities, breeding methods, types of varieties developed and disseminated, and genetic knowledge generated, as well as notable missteps and lessons learned, as this will direct development of future strategies aimed at enhancing efficiency and impact robusta breeding.

### 2.1 Breeding in Indonesia

The first robusta breeding program was initiated by scientists based in Dutch East Indies (now known as Indonesia), which was the first recipient of robusta germplasm of African origins (Ferwerda, 1948; Cramer, 1957). Three institutes started breeding programs following their acquisition of robusta germplasm: Besoekisch Proefstation (precursor to the Indonesian Coffee and Cocoa Research Institute [ICCRI]) in Jember, and its affiliate Kaliwining Experimental Station, primarily utilized the introduced germplasm to develop the renowned Besoekisch Proefstation (‘BP’) variety series. Proefstation Midden Oost Java in Malang utilized the introduced germplasm to develop the renown Soember Asin (‘SA’) variety series. Finally, the Government’s Experimental Farm in Bangelan, Malang, utilized the introduced germplasm to develop the renown ‘Bgn’ variety series (Baon, 2011).

Priority breeding traits in these institutes included enhancing the regularity of fruit bearing, cherry yield stability, bean size, outturn (ratio of weight of harvested and clean coffee cherries to the weight of processed green coffee beans), plant architecture, and resistance to diseases and insect pests (Ferwerda, 1948). As a pioneer breeding program, adjustments in methods were commonplace. For example, the period between 1907 and the 1930s saw experimentation involving mass selection, row-progeny testing, use of inbreeding, and the introduction of cloning and/or grafting (Ferwerda, 1948). Because of the long history of robusta breeding in Indonesia, it is conceivable that different phases of their breeding efforts have yielded distinct sets of varieties, each reflecting the rationale for its development and breeding strategies used to produce them (**Table 2**).

**Table 2:** Variety development and replacement in Indonesia from 1920 to 2019.

Breeding period	Breeding institute	Varieties released	Breeder notes
1920 to 1940	Government’s Experimental, Bangelan, Malang	‘R.Bgn.124’, ‘R.Bgn.124.01’, ‘Q.Bgn.121’, ‘Rob.Bgn.371’, and ‘Bgn.300’	‘R.Bgn.124.01’ is an outstanding rootstock
	Proefstation Midden Oost Java in Malang	‘SA 13’, ‘SA 34’, and ‘SA 158’	
	Kaliwining Experimental Station, Jember	‘BP 4’, ‘BP 25’, ‘BP 39’, ‘BP 42’, and ‘BP 56’	BP 39’ and ‘BP 56’ were excellent for vegetative and multiplication (high yielding)
1940 to 1970	Indonesian Coffee and Cocoa Research Institute (ICCRI)	Hybrid variety derived from ‘BP 42’ × ‘BP 358’ widely cultivated	Bi-parental crosses adopted as a breeding strategy
1997 to 2005	ICCRI	‘BP 234’, ‘BP 288’, ‘BP 358’, ‘BP 409’, ‘BP 308’, and ‘SA 237’	Varieties destined for arid regions; ‘BP 308’ is highly resistant to nematodes
2010 to 2019	ICCRI	‘Sintaro 1’, ‘Sintaro 2’, ‘Sintaro 3’, ‘Sehasence’, ‘Korolla 1’, ‘Korolla 2’, ‘Korolla 3’, ‘Korolla 4’, ‘Basemah 1’, ‘Basemah 2’, ‘Basemah 3’, and ‘Basemah 4’ “Libtukom”	Outstanding farmer-led selections officially released. “Libtukom”, is a synthetic *C. liberica* destined for peatland areas
After 2019	ICCRI	‘Hibiro 1’ (‘BP 936’ × ‘BP 534’), ‘Hibiro 2’ (‘BP 534’ × ‘BP 936’), ‘Hibiro 3’ (‘BP 939’ × ‘BP 936’), ‘Hibiro 4’ (‘BP 935’ × ‘BP 436’), and ‘Hibiro 5’ (‘SA 13’ × ‘BP 436’)	Previously released varieties used as progenitors

Since 1992, the Indonesian government has implemented regulations mandating the Ministry of Agriculture to certify planting material to safeguard farmers from accessing inferior seed.

As the first breeding program for robusta, much of the knowledge generated was foundational, while the techniques developed were transformative. First, ease of propagation was considered an important selection trait. Accordingly, the suitability of a variety for commercial production was assessed based on ease of vegetative propagation; ease of seed production; and ease of both seed and vegetative propagation. Second, the concept of hybrid and/or synthetic varieties emerged from these early efforts, hence adding the consideration of ‘biclonal’ and/or ‘polyclonal’ seed gardens. While both ‘biclonal’ and ‘polyclonal’ seed gardens aim to produce genetically superior cross-pollinated seed for commercial production, the former involves only two compatible and complementary clones, while the latter often is no less than three compatible and complementary clones planted in a randomized or systematic layout to maximize cross pollination. Although there was no consensus on the number of clones to include in a ‘polygarden’, important considerations were and remain genetic diversity, trait blending, pollination efficiency and seedling quality.

Third, inter-specific hybridisation was exploited, such as with the generation of hybrids derived from crosses between *C. canephora var ugandae* and *C. congensis* called ‘Congusta’ hybrids (Ferwerda, 1948). Fourth, the registration and official release of varieties selected by farmers following conduction of performance trials (Ramadiana et al., 2018; Syafaruddin et al., 2020). Overall, the breeding programs in Indonesia have pursued continuous improvement, adopting improved methodologies to drive further genetic gains. Robusta breeding experiences and knowledge generated in Indonesia were applied elsewhere.

### 2.2 Breeding in India

The devastating consequences of coffee leaf rust (CLR) on arabica in India drove the import of robusta germplasm from Indonesia to the Wayanad district of the Indian State of Kerala in the early 1900s. *Coffea liberica* was also introduced at this time (Prakash et al., 2016). Additional germplasm resources later originated from Uganda, Madagascar, Ghana, and the Ivory Coast (Vishveshwara, 1970; Prakash et al., 2016). Later, the robusta variety ‘Paradeniya Robusta’ was introduced from Sri Lanka. This introduced robusta was later renamed ‘Old Robusta’, purportedly to distinguish it from other modern robusta selections. The ‘Old Robusta’ remains one of the dominant robusta cultivars in India due to its broad adaptability, vigorous growth, and good cup quality.

The huge popularity of ‘Old Robusta’ made it a benchmark variety and a basis for robusta breeding in India. Breeding priority traits included high yields, enhanced adaptability to different environments, greater bean size, good cup quality, and compact stature. Three robusta varieties that had these priority traits were developed and released for commercial cultivation by the Central Coffee Research Institute (CCRI), the main research institute under the Coffee Board of India. The development strategies employed are outlined in Prakash et al. (2016).

Briefly, in the 1940s, the variety ‘Sln. 1R’, was developed. The variety is deployed as seedling progeny generated through hybridisation of two high-yielding mother plants. This variety exhibited better cup quality and bean size, with broad adaptability across most robusta-growing regions. Two decades later, the variety, Sln. 2R–Balehonnur Robusta’ (also referred to as ‘Sln.2R’) was released owing to its improved cup quality, uniform yield, and bean size as compared to ‘Sln. 1R’. This variety is derived from three high-yielding clonal varieties (BR series 9, 10 and 11). In the 1970s, ‘Sln. 3R’ was released owing to its enhanced cup quality and compact bush stature as compared to other previously released and/or commonly grown varieties. Suffice to note that ‘Sln. 3R’ is a derivative of an inter-specific cross between *C. congensis* and *C. canephora* with recurrent backcrossing to *C. canephora.* The three varieties (Sln, 1R, Sln. 2R and Sln. 3R) represent the foundation of India’s Robusta breeding program, as they are all adaptable to all robusta-growing regions in India. **Table 3** provides a summary of attributes associated with these robusta releases in India.

**Table 3:** Varieties developed and disseminated in India.

Selection	Key traits	Breeder notes
Sln. 1R (S.274)	Yield 2,000–2,500 kg/ha under irrigation Yield 1,000–1,500 kg/ha without irrigation Medium sized beans ~ 45% ‘A’ grade, with good cup quality Wide adaptability	Seedling progenies of two high-yielding mother plants
Sln. 2R (BR Series 9, 10, 11)	Growth habit, yield potential and cup quality similar to ‘S.274’ High stability for production and grade A beans Wide adaptability	High-yielding clonal progenies (‘BR 9’, ‘BR 10’, ‘BR 11’)
Sln. 3R (CxR hybrid)	Compact with bush stature Early and uniform bearing Yields 2,000–2,500 kg/ha under irrigation Yields 1,000–1,500 kg/ha without irrigation High mucilage and thus easy pulping Suitable for planting at closer spacing (i.e., 2.7 × 2.7 m or 2.4 × 2.4 m) Medium sized beans with 50–55% ‘A’ grade with good cup quality Wide adaptability	Inter-specific hybridisation between *C. congensis* and *C. canephora;* resultant progeny backcrossed to *C. canephora*

India adheres to a structured procedure to release new varieties, which can be done at the state or national level. The Coffee Board of India (https://coffeeboard.gov.in/) is instrumental in recommending varieties for release. Both synthetic varieties (seed propagated) and clonal varieties (vegetatively propagated) have been released.

Two complementary breeding strategies are currently being pursued in India. The first strategy focuses on the development of specific clonal varieties by harnessing the standing genetic diversity present within Indian robusta germplasm collections. This strategy aims to develop new varieties following five steps: 1) positive identification of elite clones with desired trait combinations in the available germplasm including materials in farmers’ fields; 2) performance evaluation of selected mother clones over at least five years; 3) performance ranking of mother plants and assessment of clone compatibility; 4) propagation of compatible mother plants; and 5) conducting multi-locational evaluation field trials tailored toward the release of outperforming clonal sets.

The second strategy focuses on intra-specific and inter-specific hybridisation, as this has made remarkable breakthroughs via exploiting inter-specific hybridisation between *C. canephora* and *C. congensis* (Prakash et al., 2016). Notably, India has adopted unique eco-friendly practices, cultivating robusta in the shade and/or with intercropping, that enhance quality profiles to meet market needs (ICO, 2024, Prakash, 2018).

### 2.3 Breeding in Vietnam

Robusta was largely introduced into Vietnam from Congo and/or Indonesia by the French in the early 1900s. In fact, the genetic relationships between Vietnamese and Ugandan populations can be traced back to the Congolese genetic group (Garavito et al., 2016; Vi et al., 2025). The point of introduction in Vietnam was the Central Highlands region, from where robusta spread to other regions. Records indicate that formal robusta breeding commenced in Vietnam in the late 1970s, mainly by the Western Highlands Agriculture and Forestry Science Institute (WASI), whose objectives were to develop and deploy varieties for commercial production in Central Highlands region (Phan, 2017).

Although published information on robusta breeding in Vietnam is unavailable, it’s evident that over the past five decades, both early and late-maturing varieties have been officially released and disseminated to growers. These varieties are characterized by rust resistance, high yields, and high bean quality. Some of the most outstanding clonally propagated varieties include ‘TR4’, ‘TR9’, ‘TR11’, ‘TR12’, ‘TR14’. and ‘TR15’, while ‘TRS1’ is a seed-propagated variety that was selected following hybridisation among four clones, TR4, TR9, TR11, and TR12 (Phan, 2017). Recent varieties, including TR14 and TR15, have been released specifically to cope with climate change. Farmers within Vietnam can only access and plant seeds provided by WASI.

### 2.4 Breeding in Uganda

Uganda was one of the origins of robusta; in particular, the Congolese genetic group. This suggests that local farmers have made informal selections to adapt coffee from the wild for several centuries (Kiwuka et al., 2021; Davis et al., 2023). These local selections yielded the widely cultivated and renowned ‘Nganda’ type of robusta varieties (Thomas, 1935). This is a world-class example of leveraging local genetic diversity to solve local agricultural problems, as it moved robusta from the wild and adapted it to commercial production under low-input farming systems.

Seeds introduced into Uganda in 1910 from Indonesia separately gave rise to the ‘Erect’ (or Erecta) robusta type of varieties (Thomas, 1935). The ‘Erect’ robusta type is characterized by upright stature, compact, faster growth, early fruition, and more yielding than individuals from the Nganda robusta type that primarily are characterized by a bushy and spreading growth habit. These robusta types often intermix without restrictions and thus enrich the genetic diversity of Uganda’s robusta population, as revealed in the study by Kiwuka et al. (2021).

Pioneer breeding was conducted in the early 1900s at Kawanda Agricultural Research Station (Thomas, 1935; Kibirige-Ssebunya et al., 1993). Priority traits included vigor, resistance to prevalent pests and diseases, yield, bean size, and cup quality. Mass selection and the use of progeny tests were the primary breeding strategies employed during the 1930s, 1940s, and 1960s, resulting in the selection of several elite clones for cultivation (Thomas, 1935; Leakey, 1970). Elite mother trees from farmers’ fields and from forest populations were also selected for cultivation and/or for breeding. These selections often referred to as ‘elite clones’ dominated robusta production until the early 1990s when coffee wilt disease (CWD), caused by the ascomycete *Fusarium xylarioides*, devastated most of the commonly grown varieties (Musoli et al., 2013).

This outbreak stimulated concerted efforts to breed for resistance to CWD, a disease that has until now primarily been restricted to Africa (Rutherford, 2006). The adopted strategy involved evaluating available germplasm including elite clones alongside their progenies for CWD resistance (Musoli et al., 2003, 2013). Additionally, half-sibs from various wild clones and cultivated robusta (Nganda and Erect types) were tested for CWD resistance (Musoli et al., 2013). Up to 10 ‘Kituuza Robusta’ (‘KR’) varieties exhibiting CWD resistance were eventually released, largely to contain the CWD epidemic. The KR 1–7 varieties were released in 2009, while the KR 8–10 varieties were released in 2017. These KR varieties and elite clones (selections from native diversity) are both currently used for commercial production.

Robusta breeding in Uganda is now conducted by the National Coffee Research Institute (NaCORI), a public research institute under the National Agricultural Research Organisation (NARO) of Uganda. This breeding program continues to develop new varieties to address shortfalls (such as drought and disease susceptibility), in extant varieties deployed for commercial production. Furthermore, efforts are underway to integrate DNA-based markers for quality control, alongside germplasm screening for drought and/or heat tolerance (Sseremba et al., 2023). Further, collection of indigenous coffee germplasm continues to be done from forests spread across different climatic environments: Zoka (northern Uganda); Mabira (central Uganda); Maramagambo (southwestern Uganda); Budongo (northwestern Uganda), and Kibale and Itwara (both located in western Uganda). This germplasm is being collected for conservation and for future research use. The National Seed Certification Service, within the Department of Crop Inspection and Certification, and under the umbrella of the Ministry of Agriculture, Animal Industry and Fisheries (MAAIF), is mandated with variety release, seed regulation, and germplasm imports in Uganda (Department of Crop Inspection and Certification – Ministry of Agriculture, Animal Industry and Fisheries).

In addition to variety development, the robusta breeding program in Uganda has generated important knowledge. First, they performed self-pollinations and showed that the consequences of inbreeding were not as severe as previously feared (Thomas, 1935). Selfing, which leads to greater genetic homozygosity, has breeding relevance (Ceballos et al., 2015; Ramu et al., 2017; Melchert et al., 2025). Second, of the three coffee species [*Coffea canephora*, *C. eugenioides*, and *C. liberica* (var. dewevrei)] located in Uganda (Davis et al., 2023), efforts are underway to use *C. liberica* as a rootstock for robusta deployment, particularly in drier and drought-prone regions. Third, they have generated reproducible protocols for screening for CWD resistance in both field (naturally infected) and screenhouse (controlled) settings. Finally, their work provided insights into cup quality assessments that underscored prospects in genetic enhancement of quality (Aluka et al., 2015).

### 2.5 Breeding in West Africa: Ivory Coast, Ghana, and Cameroon

A few countries in West Africa, notably the Ivory Coast, Togo, Benin, Guinea, Sierra Leone, Liberia, Gabon, Ghana, Nigeria, and Cameroon (a bridge between Central and West Africa), are considered sites of origin of robusta and specifically the Guinean group, suggesting that robusta selection by indigenous people in these countries may have taken place for centuries. Formal robusta breeding only began in the 1950s during the colonial period, highlighting how current-day varieties are only marginally different from their wild ancestors.

Importantly, on-farm production volumes in the region are small, caused by several factors (such as use of unimproved varieties, climate stress, and susceptibility to pests and disease), thus offering substantial opportunities for improvement to meet the growing regional market. While the above West African countries have long had operating agricultural research institutions, more structured robusta breeding has been done, and continues to be done at three institutions: Le Centre National de la Recherche Agronomique (CNRA) in the Ivory Coast, which was a regional robusta breeding hub; the Cocoa Research Institute of Ghana (CRIG); and l’Institut de Recherche Agricole pour le Développement (IRAD) in Cameroon. These institutions have had scientific collaboration with various partners on robusta genetic improvement.

Wild and semi-wild populations selected by local farmers prior to the 1950s displayed subpar traits and poor productivity. Global germplasm collection efforts were made between the 1950s and late 1980s, including the establishment of a gene bank in the Ivory Coast (Bramel et al., 2017). Germplasm from this gene bank was used by various West African countries for variety development (Capot, 1977; Bouharmont et al., 1986; Bouharmont and Awemo, 1979; Leroy et al., 1993; Cilas and Bouharmont, 2005; Cilas et al., 2003; Leroy et al., 2014). Other countries, such as Ghana, made additional germplasm introductions from diverse sources: from West Africa, open-pollinated seeds obtained from the Ivory Coast and clones from Togo; from Central and East Africa, clones received from Cameroon, the Democratic Republic of Congo, Uganda, and Tanzania; and from Asia, open-pollinated seeds imported from Vietnam. These introductions were used as founders for robusta breeding in Ghana (Akpertey et al., 2022a).

Across West Africa, the priority traits considered during breeding included high yield, resistance to prevalent pests and diseases, drought tolerance, plant architecture, ease of processing, cup quality, and ease of propagation. Phenotypic mass selection was the indispensable first strategy that allowed West African Robusta breeding programs to begin the transformation of coffee by selecting locally adapted varieties from wild, unselected populations. Later, the reciprocal recurrent selection (RRS) implemented in Ivory Coast, was adopted to harness traits inherent within “Guinean” and “Congolese” gene pools (Leroy et al., 1993). Principally, RRS aimed at generating improved hybrid performance through simultaneous improvements of both Guinean and Congolese gene pools as this is important when breeding for both additive traits (such as tree height, stem diameter, bean size and laterals per tree) and non-additive traits (such as vigor, flowering synchronization, cup quality, stress tolerance, disease resistance and cherry yield).

These breeding efforts yielded three key findings: 1) the progeny of crosses between different groups (inter-group progeny) reached yields between 16% and 140% relative to the mean of two clonal commercial varieties (461 and 126), when evaluated on a single-plant basis (Leroy et al., 1997); 2) consistent positive correlations (r >0.55) were recorded between early vigor and yield (Leroy et al., 1997); and 3) testers, which principally evaluate genetic merit of unknown individuals, were reliably used to predict yield performances of 78 Congolese and 100 Guinean clones; as robusta is outcrossing, heterozygous testers proved valuable in assessing genetic merit of individuals (Montagnon et al., 2007).

Similarly, other key findings from robusta breeding in the Ivory Coast were: 1) narrow-sense heritability for yield based on single-tree plots, evaluated over four years, ranged from 0.25 to 0.45 (Montagnon et al., 2003); 2) that doubled haploid (DH) individuals can be crossed to either heterozygous genotypes or other DH clones, leading to pronounced hybrid performance (Lashermes et al., 1994); 3) hybridisation campaigns between *C. canephora* and *C. liberica* helped enhance resilience and productivity (Yapo et al., 2003), while crosses between *C. canephora* and C*. congensis* enhanced cup quality (Ouattara et al., 2021); and 4) vegetatively-propagated varieties outperformed their seed-propagated counterparts (Capot, 1977). These findings are of relevance when designing new robusta breeding strategies that entail progenitor selection, optimization of field trialing, utilization of other coffee species for enhanced robusta performance, and dissemination of new improved varieties to farmers.

Insights on genetic erosion within robusta have also been made in Ivory Coast; this study was prompted by the vast introductions of Congolese accessions in the preceding decades, which were thought to pose a threat to the native wild Guinean group (Gnapi et al., 2022). The study involved field observations of flowering patterns and genotyping of open-pollinated Guinean, Congolese, and their respective hybrid offspring for paternity analysis. There is an urgent need to conserve wild Guinean populations in West Africa, as the study found that Congolese pollen grains are more effective at pollinating the Guinean group than Guinean pollen on Congolese trees (Gnapi et al., 2022).

Robusta breeding in Ghana provided more valuable insights, including:1) selecting genotypes based on average cherry yields over the first three years of a seven-year experiment was about 75% efficient relative to the seven-year average yield (Anim-Kwapong et al., 2011); 2) juvenile traits (i.e., number of laterals and trunk cross-sectional area) are predictors (r = 0.24 to 0.57) for cherry yield at later stages (Akpertey et al., 2019); and 3) single nucleotide polymorphism markers can be used reliably during breeding, for example to verify parental identity and help flag mislabeled germplasm plots (Akpertey et al., 2021).

Cameroon’s unique location (a link between Central and West Africa) suggests that it is naturally endowed with extensive genetic diversity that comprises both Guinean and Congolese individuals, and their respective hybrid progenies, making it a key reference for robusta genetic diversity (Gomez et al., 2009; Montagnon et al., 2012). Accordingly, phenotypic mass selection was the main breeding strategy used then to adapt robusta from the wild to new production environments with primary focus on yield and agronomic traits.

One of the most significant studies conducted in Cameroon was the 14-year study that examined genetics of bean traits (normal bean weight, the percentage of pea berries, the length, width and thickness of the beans) alongside yield (Cilas and Bouharmont, 2005). In that study, yield was the most heritable trait (H^2^ = 0.43), as compared to bean size and its dimensions with H^2^ ≤0.1, while negative and significant genetic correlations were consistently observed between yield and peaberry rate (Cilas and Bouharmont, 2005).

A related study examined the rate of peaberry development in robusta clones sourced from different countries (clones B5, B11 and B42 from the Republic of Central Africa, clones C5 and C6 from Ivory Coast, clones J13 and J21 from Java, and clone M5 from Madagascar). At one of the evaluation sites (Nkolbisson, central Cameroon), pea berry rates ranging between 35 and 60% were observed (Fallo and Ngongang Nono, 2005) in clones C5, J13, J21 and M5, underscoring the need to reduce peaberry rates, as this affects both quality and quantity of beans. Selecting for low peaberry production, high compatibility and/or high pollination efficiency should be able to reduce peaberry rates.

The above breeding efforts in the Ivory Coast, Ghana and Cameroon, have resulted in the release of seed-propagated (synthetic and hybrid) and vegetatively-propagated varieties which once accessed and planted in farmers’ fields could enhance production and productivity not only in these countries but also the entire West African region (Bouharmont and Awemo, 1979; Anim-Kwapong et al., 2011; Akpertey et al., 2020, 2022a). The growing and/or emerging coffee markets in West Africa are a motivation for further robusta innovation in the region that will benefit from partnerships with other institutions involved in robusta research and development.

### 2.6 Breeding in Brazil

*Coffea canephora*, popularly known in Brazil as conilon (*C. canephora* var. *conilon*) or robusta (*C. canephora* var. *robusta*), was introduced in the state of Espírito Santo in the early 1900s. As mentioned above, conilon trees share phenotypic characteristics with individuals from the Congolese and Guinean genetic groups, raising the possibility that conilon may have resulted from a cross between these genetic groups prior to its introduction in Brazil. Since its introduction, significant expansion only happened after the 1970s, after production challenges were encountered with *C. arabica*, notably outbreaks of CLR and frost damage in 1975 (Zambolim et al., 2024). The cultivation of robusta clones originally developed in Espírito Santo expanded locally, driven by the migration of farmers away from the area. Farmer migrations have in fact greatly facilitated germplasm movement, expanded production, and helped generate new genetic variation (Ferrão et al., 2019; Silva et al., 2025). Technological advances (such as irrigation and improved climate-resilient varieties) and growing market demands, all contributed to robusta expansion beyond Espírito Santo.

There are five main federal and/or state level institutions that have been involved in undertaking collaborative robusta breeding operations in Brazil: 1) Instituto Agronômico de Campinas (IAC), São Paulo; while historically renowned for arabica research, it contributed to early robusta introductions, trait discovery and pre-breeding; 2) Universidade Federal de Viçosa (UFV), Minas Gerais; one of the academic public universities renowned for its contribution towards undertaking empirical studies on robusta genetic diversity assessments, drought tolerance, coffee leaf rust, and development of breeding methodologies;

3) Instituto Capixaba de Pesquisa, Assistência Técnica e Extensão Rural (Incaper), Espírito Santo; this was the pioneering institution for conilon breeding and renowned for development of outstanding clonal and seed propagated varieties, including the development of polyclonal seed garden approaches; 4) Universidade Federal do Espírito Santo (UFES), Espírito Santo; an academic institution that has collaborated closely with Incaper in training students and conducting genetic studies; and 5) Empresa Brasileira de Pesquisa Agropecuária (Embrapa, Rondônia); a federal research institution that coordinates all coffee research. However, Embrapa Rondônia, conducts state-level research and focuses on breeding both conilon and robusta types destined for the Amazonian region.

Strong institutional collaboration is evident in Brazil. For example, the Incaper and Embrapa research collaboration entails sharing germplasm and conduction of joint evaluation trials. On the other hand, private entities including private nursery operators and cooperatives, are involved in multiplying and selling varieties developed by the public institutions (Incaper and Embrapa). The main coffee research initiatives in Brazil are coordinated by the Consórcio Pesquisa Café (CPC), which implements research projects under the National Coffee Research and Development Program (Programa Nacional de Pesquisa e Desenvolvimento do Café [PNP&D/Café]).

The robusta germplasms collected and conserved at these institutes, alongside trees kept in genebanks and in farmer fields, formed the base populations for breeding (Senra et al., 2020; Zambolim et al., 2024). Most Brazilian breeding programs initially relied on the use of phenotypic mass selection and thus generated appreciable genetic variation. This diversity was meticulously exploited by Incaper to develop outstanding varieties that were widely adopted in the Espírito Santo. Some notable examples include ‘Diamante ES8112’, ‘Jequitibá ES8122’, ‘Centenária ES8132’, and ‘Marilândia ES8143’ (Ferrão et al., 2019, 2024). These varieties were developed after 20 years of experimentation involving over 2,000 clones (Ferrão et al., 2019). Embrapa (Rondônia) has equally developed and released robusta varieties specifically for production in Western Amazon; these varieties are selected for enhanced yield, resilience to CLR and root-knot nematodes, beverage quality and compatibility (Teixeira et al., 2020). Over the years, the uncontrolled coexistence of “conilon” and “robusta” plants led to the emergence and cultivation of varieties with intermediate traits between the two groups (Oliveira et al., 2018; Rocha et al., 2021). Indeed, a recent study that characterized coffee trees grown in Amazonia, confirmed a significant number of hybrids mostly generated between robusta and conilons, that were selected and grown by farmers (Silva et al., 2025).

Priority breeding traits in Brazil included high productive capacity and bean quality; uniformity of fruit maturation; resistance to pests and diseases; resilience to high temperatures, drought, soils with low fertility and high levels of aluminium; amenability for mechanization, different cropping systems and altitudes; enhanced cup quality; and biennial production (Ferrão et al., 2019, 2024). Both registered varieties (**Table 4**) and farmer-selected varieties are commercially cultivated (Teixeira et al., 2020). These varieties combine desirable agronomic traits, such as vegetative vigor, high yield, disease resistance, and drought tolerance (Espindula et al., 2022). Also noteworthy is pre-breeding done at Instituto Agronômico de Campinas that led to the development of the robusta clone Apoatã IAC 2258 that was used extensively as a nematode-resistant root stock to revitalize coffee production in nematode-infested areas particularly in São Paulo, Minas Gerais, Espírito Santo in Brazil (Carbonell et al., 2012). Taken together, all these efforts have thus been pivotal in sustaining robusta production and productivity in Brazil.

**Table 4:** A selection of robusta varieties developed and disseminated in Brazil.

Holder	Cultivars
Incaper	‘Conilon’, ‘Emcapa 8131’, ‘Emcapa 8121’, ‘Emcapa 8111’, ‘Emcapa 8141’, ‘Emcaper 8151’, ‘Vitória Incaper 8142’, ‘Centenária ES8132’, ‘Diamante ES8112’, ‘Jequitibá ES8122’, ‘Marilândia ES8143’, ‘Conquista ES8152’, ‘Goytacá ES8161’
Embrapa	‘BRS Ouro Preto’, ‘BRS 2314’, ‘BRS 3220’, ‘BRS 3213’, ‘BRS 3137’, ‘BRS 3210’, ‘BRS 1216’, ‘BRS 2336’, ‘BRS 3193’, ‘BRS 2357’, ‘BRS 2299’, ‘BRS Primalta’, ‘BRS Primalta 20’, ‘BRS Primalta 30’, ‘BRS Primalta 40’, ‘BRS Primalta 50’, ‘BRS Primalta 60’, ‘BRS Primalta 70’, ‘BRS Primalta 80’, ‘BRS Primalta 90’
UFES	‘Tributun’, ‘Monte pascoal’, ‘Salutar’, ‘Forte Guarani’, ‘Plena’
IAC	‘Apoatã IAC 2258’, ‘IAC Herculândia’
Procafé Foundation	‘Colatina PR6’
IFGoiano/UFES	‘Andina’
UENF/UFES	‘Magnus Grano’
Natural person	‘Ipiranga 501’, ‘SV 2010’, ‘Verdebrás G30/G35’

The National Register of Cultivars (Registro Nacional de Cultivares [RNC]), supervised by the Ministry of Agriculture, Livestock and Supply (MAPA), serves as the official system for registering new plant cultivars. Registered varieties must meet standards for genetic identity, agronomic performance, and commercial viability. Both synthetic varieties (seed propagated) and clonal varieties (vegetatively propagated) have been released.

Incaper is arguably the most important conilon breeding institution in the world. Over the years, Incaper has documented 10 steps, taking 12 years to complete, leading to the development of clonal cultivars that are released and deployed in sets (Ferrão et al., 2019). These 10 steps are: 1) identification of higher-performing and diverse individuals (years 1–3); 2) vegetative propagation of selected individuals (year 3); 3) experimental evaluation of selected clones in comparative trials over four harvests (years 3–10); 4) statistical analysis of empirical data to inform decisions and/or selection choices (years 5–12); 5) assessment of beverage quality (year 8); 6) vegetative propagation of outstanding clones and grouping (years 8, 9); 7) genetic compatibility tests based on pollination efficiency and fruit set (years 9, 10); 8) registration of new varieties for certificate of protection (years 11, 12); 9) multiplication of the superior clones in clonal gardens (years 11, 12); and 10) variety release and launch (year 12).

Robusta breeding in Brazil has over the years generated foundational knowledge and transformative techniques. First, pre-breeding conducted in the 1950s at IAC transferred CLR resistance genes from robusta to arabica varieties, which helped revive the Brazilian coffee industry 20 years later, in the 1970s, when arabica was devastated by CLR epidemic (Carbonell et al., 2012; Zambolim et al., 2024).

Second, synthetic varieties like ‘Emcaper 8151-Robusta Tropical’ were released by Incaper, targeting small-scale and/or medium-scale farmers who often lack the resources to access clonally propagated varieties. Third, reproductive compatibility among clones was considered when deploying varieties (Santin et al., 2019; Silva et al., 2024). Indeed, cultivating self-incompatible plants, in which fruit production requires outcrossing with different compatibility clones, tends to be less successful when ≥ 6 clones of different compatibility groups—and thus different alleles of the self-incompatibility *S* locus—are planted together (Silva et al., 2024).

Fourth, tools like near infra-red spectrometry that are used to measure biochemical and physiological traits in non-destructive ways (Santos et al., 2012) and genomic selection were developed and implemented to enhance breeding (Adunola et al., 2023, 2024; Rocha et al., 2025) and overcome breeding obstacles associated with phenotypic trait evaluations. Inroads have also been made towards the development of specialty robusta that entails definition of sensory, physical and processing criteria to be adopted (Agnoletti et al., 2024; Viencz et al., 2023). From the aforementioned achievements, designing new robusta breeding strategies elsewhere will undoubtedly benefit from the fundamental knowledge and techniques generated and/or developed in Brazil.

### 2.7 Other breeding initiatives

In addition to the breeding efforts highlighted above, both formal and informal robusta breeding have taken place in other places, although published details on these programs are limited. For example, farmer-led selection at likely robusta birth places in northern Angola (Vos, 2021), Democratic Republic of Congo (Bopoko et al., 2024) and northern Tanzania (Ng’homa et al., 2017), is likely to have occurred and continues to occur to meet local production and marketing needs. At the same time, formal breeding has and continues to be led by other private and public institutions operating in Africa, Asia, and Latin America. The following section offers a summary of some of these institutions involved in formal robusta breeding, based on available public information.

#### 2.7.1 Breeding at Nestlé

Nestlé, the world’s largest coffee company, has been pursuing robusta breeding for several years. The breeding program initiated by Nestlé emphasizes the development of robusta varieties that are high-yielding, resilient to both higher temperatures and longer dry seasons, more resistant to pests and diseases, and exhibiting high cup quality. Field breeding operations are mostly located at experimental farms in Ecuador, Thailand, and the Ivory Coast, as well as at different local partner sites. Robusta clones from Nestlé have been distributed to selected countries and are currently being grown in Central America https://www.nestle.com/aboutus/research-development/news/nestle-scientists-discover-unique-low-carbon-drought-resistant-coffee-varieties.

#### 2.7.2 Breeding at CIRAD

The French Agricultural Research Centre for International Development (Centre de coopération internationale en recherche agronomique pour le développement [CIRAD]) has made significant contributions to robusta breeding. Indeed, CIRAD pioneered breeding activities, particularly in West Africa and specifically in the Ivory Coast and Cameroon, where CIRAD worked in collaboration with CNRA and IRAD as described earlier. In East Africa, specifically in Tanzania, CIRAD in a partnership with the Tanzania Coffee Research Institute (TaCRI), supported the development and dissemination of improved arabica and robusta varieties (Kilambo et al., 2015).

CIRAD continues to play a role in robusta breeding, especially in Africa and Asia, with a strong focus on genetic improvement, climate resilience, and agroforestry systems, as highlighted in both the ROBUST (https://en.ird.fr/project-robust-growing-robusta-coffee-agroforestry-adapt-and-mitigate-climate-change-uganda) and the BREEDCAFS (https://www.cirad.fr/en/cirad-news/news/2022/breedcafs-for-the-coffee-of-the-future) projects. Briefly, the ROBUST project seeks to counteract the negative impact of climate change by exploiting agroforestry, germplasm diversity, and variety development. On the other hand, BREEDCAFS aims at breeding varieties for agroforestry systems; Vietnam and Cameroon are some of the robusta-producing countries involved field trialing components of this project.

#### 2.7.3 Other countries with robusta breeding activities

Among other public breeding programs, we highlight examples to underscore the growing interest in robusta within and beyond its sites of origin. In Kenya, a predominantly arabica producing country, efforts are underway to develop and commercialize “arabusta” hybrids, which are inter-specific crosses between *C. canephora* and *C. arabica* (Cheserek et al., 2020). The Tanzanian Coffee Research Institute (TARI) located in northern Tanzania, is rejuvenating coffee through undertaking demand-driven research to address key production constraints; phenotypic mass selection has been the main strategy to deploy and deploy new robusta varieties that combine high-yield, coffee wilt disease resistance with enhanced cup quality (Kilambo et al., 2015; Mware et al., 2020; Kihara et al., 2021).

Even though the DRC is one of the origins of robusta, and more specifically the Congolese genetic group, it is significant that in the early 1900s, robusta selections from Indonesia were brought back to the DRC at the National Institute of Agronomic Research ((INERA), Yangambi, Tshopo province, for dual roles of breeding and germplasm conservation. The dearth of published information on breeding activities in DRC makes it challenging to highlight the breeding operations and/or activities that have been conducted since then, regardless of the protracted civil conflict that the nation has gone through and continues to witness.

Nonetheless, it is clear that many of the INERA-Yagambi collections (which are mostly admixed) predominate materials grown in farmers’ fields (Abeele et al., 2021), suggesting that phenotypic mass selection for stress resilience, productivity, and adaptability may have been the primary selection strategy used at the time. Currently, efforts are underway to enrich the Yangambi collections with under-represented wild accessions and to systematically utilize the native genetic diversity for breeding (Verleysen et al., 2023). Indeed, selections from the Yangambi rehabilitated germplasm collections have been made with plans to utilize both wild and cultivated robusta for breeding (Bollen et al., 2024a, 2024b). Foundation-laying phase is also underway in Angola to revamp robusta production and productivity; this is being attained by leveraging both unique adapted landraces and introduced germplasm. In Latin America, robusta production and research are taking place in Mexico (Garavito et al., 2016), and prospects for robusta production and breeding are also being explored in Colombia (González-Orozco et al., 2024) and Ecuador (Loor Solórzano et al., 2017) alongside Nicaragua. Thailand and Philippines are additional Asian countries with higher prospects for robusta innovation.

Each of the breeding efforts highlighted above provides important knowledge that can readily be adopted by other breeding programs henceforth. For example, the biclonal and polyclonal seed garden setups adopted in Ghana, Ivory Coast, Indonesia, India, Vietnam and Brazil, can sustain large-scale seed production while maintaining genetic diversity, a lesson highly relevant for countries facing seed supply bottlenecks. From both the African and Asian breeding programs, we further learn how they harnessed traits inherent in the different genetic groups and/or other coffee species to enhance robusta productivity.

World-class examples of how farmer-selected clones can finally be formally released for commercial cultivation are Brazil and Indonesia. Additionally, Brazil offers exceptional illustrations of the advantages of applying tried-and-true breeding innovations (like genomic selection, near infra-red spectrometry, rootstock technology deployment, and clone compatibility knowledge) to improve breeding impact and efficiency.

Inbreeding exploitation, as explored in Indonesia and Uganda, was not definitive and might call for more investigation. The need for pre-emptive breeding is further supported by the fact that some economically relevant diseases and pests like strains of *Fusarium xylarioides* (causing coffee wilt disease), *Cercospora coffeicola* (causing coffee red blister disease), and species of *Leucoptera coffeella* (coffee leaf miner), and nematode species, are currently restricted to specific geographies but have a high likelihood of spreading to other areas as a result of climate change.

### 2.8 Summary of evolving traits, breeding methods and varieties developed

The initial focus of robusta breeding aimed at maximizing agronomic efficiency, plantation sustainability, and industrial suitability, but not cup quality, at both the origins in Africa, where robusta was introduced from the wild, and in other regions (like Asia and Latin America), where robusta was introduced into human-modified environments. Notable traits selected for aimed at attaining: a) high yields, which guaranteed economic viability; b) resistance to locally prevalent pest and diseases, which guaranteed stable production; c) climate adaptability to different environments and altitude, which ensured rapid wide geographic spread; and d) tree vigor and longevity, which sustained production without need for frequent replanting. Selection for these traits was made easier by robusta’s out-crossing nature, which allowed for the creation of sufficient genetic variation.

It suffices to note that selection priorities for *C. canephora* were slightly different from those of *C. arabica*, which during early attempts besides selection for production-enhancing traits concentrated on: a) exclusive adaptation to highland environments, and b) enhanced cup quality. The ever-increasing robusta cultivation since 1990s has resulted into new traits requirements such as: a) enhanced cup quality, b) plant architecture compatible to farming systems, c) resistance to newly emerging and/or more virulent pests and diseases (like coffee wilt disease in Africa, and root-knot nematodes in Asia and Latin America), and d) increased resilience to abiotic stresses notably drought, heat and salinity tolerance. We anticipate continued re-ranking of trait priorities and thus the need for continued feedback from both farmers and industry, to update new trait priorities and thus guide future robusta breeding.

Guided by the traits, breeding programmes across the globe implemented breeding methods that were largely adopted from Indonesia. A few tactical changes were made in Indonesia between 1900 to 1940s including use of: phenotypic mass selection, row-progeny testing, prospects for inbreeding, and the introduction of grafting (Ferwerda, 1948). By far, phenotypic mass selection was the predominant breeding method adopted (Thomas, 1935; Prakash et al., 2016; Oliveira et al., 2018; Rocha et al., 2021; Silva et al., 2025). Reciprocal recurrent selection was only started in the early 1980s (Leroy et al., 1993) and hasn’t been systematically pursued further. Use of doubled haploids was evaluated (Lashermes et al., 1994) but has since then not been pursued further. Recurrent selection with or without integration of genetic markers is increasingly becoming a preferred breeding method to adopt (Alkimim et al., 2021; Campuzano-Duque and Blair, 2022). From these breeding efforts, two main variety types have been developed and disseminated; these are “seed propagated varieties” and/or “clonally propagated varieties” as highlighted in **Tables 2**–**4**.

### 2.9 Summary on germplasm collection, conservation and utilization

Countering the loss of genetic resources and its use for genetic improvement are some of the main motivations for germplasm collection and conservation. Accordingly, two configurations are evident for robusta. First, collection missions and conservation efforts within the African origins, and second, outside the origins. Within the African origins, collections, conservation and utilization of robusta germplasm continues to be undertaken both formally (institutional-led) and/or informally (farmer-led). Majority of germplasm collected is preserved in *ex situ* gene banks (which, despite their fragmentation, offer controlled access), *in situ* forest reserves (which facilitate further natural evolution), and in farmer fields (which facilitate gene flow “cultivated-to-wild” or “cultivated-to-cultivated” alongside identification of locally adapted individuals).

For example, in Uganda, one of the origins for “Congolese” genetic group, excursions are often undertaken in forests and in farmer’s fields to collect wild and/or feral germplasm for pre-discovery research, breeding and/or conservation (Kiwuka et al., 2021; Davis et al., 2022). Relatedly, in Democratic Republic of Congo, underrepresented wild accessions of “Congolese” genetic group are often collected to enrich gene bank collections (Abeele et al., 2021; Verleysen et al., 2023, 2024). Similar activities continue to be undertaken in other parts of central and eastern Africa and in other uniquely placed origins in southern Africa notably Angola and Madagascar.

In West Africa, another robusta origin and specifically for “Guinean” genetic group, both institutional-led and/or farmer-led collections have historically been done ‘within” and “between” countries including Ivory Coast, Guinea, Togo, Nigeria, Cameroon, and Ghana, with Ivory Coast playing a central role (Leroy et al., 2014; Akpertey et al., 2022b; Gnapi et al., 2022; Baba Nitsa et al., 2023). Germplasm exchange between Western, Central and Eastern African regions has also been on-going (Leroy et al., 2014; Gnapi et al., 2022). One notable challenge of this exchange is the threat to the native “Guinean” genetic group by the aggressive “Congolese” pollen (Gnapi et al., 2022). As noted earlier it’s this assembled germplasm that was eventually used as foundation for most robusta breeding efforts within Africa.

The pioneer exchange (collection and exportation of robusta germplasm) destined to Asia and/or Latin America in the early 1900s, laid a foundation for initial robusta conservation and utilization out of the African origins. While Brazil was the primary recipient of robusta germplasm in Latin America, early recipients in Asia included Indonesia, Sri Lanka, India, and Vietnam. It’s from these countries that robusta was further spread within Asia and/or within south and central America. These collections were further complemented by additional global-led collections made between 1960s and 1980s that aimed at unifying fragmented efforts on conservation and use of coffee genetic resources; these global-led collections that targeted *C. arabica*, *C. canephora* and other *Coffea* species at various origins in Africa, placed emphasis on collecting wild accession and farmer-preferred varieties (Bramel et al., 2017).

Noteworthy is that these collection missions resulted into the establishment of gene banks across the globe including those established in Ivory Coast, Democratic Republic of Congo, Madagascar, India, and Costa Rica (Bramel et al., 2017). In Brazil, robusta gene banks comprising of both robusta and conilon accessions have also been established (Ferrão et al., 2020). As noted earlier, it’s this assembled germplasm that was eventually used as foundation for most robusta breeding efforts outside the origins. A roadmap for advancing coffee genetic resource conservation and exchange has recently been proposed (Krishnan et al., 2024).

Lastly, we note that most of the genetic diversity at the origins, remains unexploited and at serious risk of disappearing. Relatedly, we notice the wild or feral populations in Madagascar that have been isolated, for a long time and thus potentially having some unique adaptation and stress traits could be a valuable resource to secure coffee cultivation worldwide. However, it is crucial to protect West African Guinean local populations from genetic erosion caused by Congolese introductions. The surviving and unexplored standing genetic variation of robusta in Angola equally deserves concerted conservation and utilization efforts.

## 3 Discussion

While the various robusta breeding programs have undoubtedly developed varieties that have enhanced production and productivity within and beyond the sites of robusta origins, breeding outstanding robusta varieties has faced and continues to face significant challenges. One exception may be Brazil, where robusta breeding innovations have, to some extent, overcome some of the most pressing challenges facing the industry (Santin et al., 2019; Silva et al., 2024; Ferrão et al., 2019; Adunola et al., 2024). Some of the noteworthy worldwide breeding challenges that are still unresolved are the long timescales needed to develop and officially release new varieties (Ferwerda, 1948; Eskes and Leroy, 2004; Ngure and Watanabe, 2024), limited access to useful genetic diversity and hence subpar tree productivity (Kiyingi and Gwali, 2012; Martinez, 2023; Bopoko et al., 2024; Ngure and Watanabe, 2024), and the misalignment between breeding pipelines and market needs (Luzinda, 2018; Ngure and Watanabe, 2024). Indeed, the innovation gap in robusta that spans genetic improvement and market positioning has been documented (Kirk et al., 2023).

The convergence of drought and heat stress in farmers’ fields presents another complex and significant breeding challenge for the 21^st^ century. Drought stress limits water availability, exacerbating oxidative damage and hence reduced photosynthesis, while heat stress accelerates water stress, denatures protein and disrupts pollination, all negatively impacting attainment of optimal yields and quality (Fahad et al., 2017). Addressing this breeding hurdle will require multi-forked and integrated approaches that will need to be validated in real-world growing environments of the 21^st^ century. Within limits of available knowledge, a few studies have offered valuable insights on how to address drought and heat stress.

Borgo et al. (2025) highlighted key transcription factors (such as dehydration responsive element binding proteins) that could guide breeding and/or nutrition-based interventions (such as use of fresh coffee husk for biochar), to mitigate these stresses. Related studies by da Silva et al. (2024) identified key traits that correlate with drought tolerance: deeper root systems, higher water use efficiency, higher leaf tissue elasticity and less pronounced leaf folding and inclination. Other options being explored include environment amelioration to reduce canopy temperatures while conserving soil moisture (Le et al., 2023), use of drought-tolerant rootstocks for grafting (Spiral et al., 2023) and use of inter-specific hybridizations (Lahai et al., 2025), which should be done urgently before some of these species become extinct. Finding practical and long-lasting solutions to these challenges should be the cornerstone of future robusta breeding programmes.

Thus, we should seize the opportunity to align and enhance breeding efforts to meet: 1) emerging farmer-preferred traits on the supply side, and 2) industry-preferred traits on the demand side that consists of processors, roasters and consumers. Progress in addressing these challenges on either side of the value chain has been slow, hindered by several technical and non-technical constraints. In this section, we outline some priority areas for improving robusta breeding efficiency and impact. We perceive breeding impact to be reflected in increased farmer profitability, reduced poverty, increased climate resilience and environmental health, all to be attained once newly developed robusta varieties are widely accessed, planted at scale by farmers and are seamlessly accepted by the industry.

### 3.1 Development of breeding populations aligned with target product profiles

Many locally selected robusta varieties have seen high adoption and widespread cultivation for decades. Notable examples include: the Nganda robusta type in Uganda (Thomas, 1935), the Kobura series of varieties in Indonesia (Wicaksono and Ono, 2022), and the introduced and locally established Old Robusta variety in India (Prakash, 2018). Importantly, these varieties were at the time all meeting farmer and market needs, underscoring societal acceptability and approval, and thus highlighting the significance of demand-led breeding. Such demand-led breeding aims to ensure that newly developed varieties achieve high adoption rates and a sustained impact, by involving key end-users during variety development to provide essential information and feedback on their trait priorities and expectations (Hussein, 2017).

To bring demand-led breeding into operation, it is crucial to first define the target product profile (TPP), which outlines a package of priority traits that new and breakthrough varieties should have as desired by stakeholders. In the case of robusta, the key stakeholders are farmers, seed entrepreneurs, processors, roasters and consumers. Ideally, breakthrough varieties should be adoption-ready with adequate off-take potential to reach farmers at scale. Breeding programs within Consultative Group on International Agriculture Research that deal with several crops (https://www.cgiar.org/), have defined three requirements for breakthrough varieties: 1) capable of having measurable impact on production and productivity at national or regional level; 2) distinctly different in terms of value and productivity compared to varieties being replaced; and 3) ready for adoption with sufficient take-off potential to reach farmers on a large scale.

Cobb et al. (2019) observes that rather than having an imaginative venture to design a variety, having a target product profile enables the breeding program to exclusively focus on the key priority traits desired by stakeholders. Surveys and focus groups are used to gather stakeholder opinions and priorities to distill priority traits that allow a breeding program to align efforts to quickly produce a new variety that meets or exceeds market expectations. This definition is vital for strategically aligning breeding programs with demand (Cobb et al., 2019; Srivastava et al., 2023; Ojwang et al., 2023). Thus, gaining deeper and more current insights into stakeholder prioritized traits (as highlighted in **Table 5**) raises the likelihood that new varieties will be valuable enough to replace older, currently dominant varieties (Collard et al., 2019; Srivastava et al., 2023; Ojwang et al., 2023).

**Table 5:** Trait packages to guide development of robusta target product profiles (TPP).

Trait categories	Trait benefit	Potential traits of emphasis	Trait priority for
Yield-potential traits	Higher and stable yields under shade-grown and/or sun-grown conditions should result in sustained higher incomes	Fresh berry yield; hundred-seed weight; phenology-related traits; and architecture-related traits	Grower, based on ***where*** and ***how*** robusta is grown
Abiotic stress traits	If not addressed, drought and higher temperatures diminish quality and yield, increase price volatility and thus hinder income growth	Flowering phenology traits, leaf physiological traits, and root-related traits	Grower, based on ***where*** and ***how*** robusta is grown
Biotic stress traits	Resistance protects yields and thus sustains higher incomes	Coffee leaf rust; coffee wilt disease; coffee berry disease; coffee red blister disease; coffee berry borer; coffee shoot borer; black coffee twig borer; and nematodes	Grower, based on ***where*** robusta is grown
Quality traits	Higher cup quality raises product market share, pricing, and thus incomes	Physical and organoleptic traits	Processor and roaster, based on ***who*** purchases, what they purchase, and ***how*** they process and consume
Processing and value chain traits	Lower processing costs and wastage resulting in higher incomes	Outturn; defects; bean density; and consistent bean size	Processor and grower, based on ***who*** purchases and ***what*** they purchase, and ***how*** they process
Propagation traits	Enable faster access to new varieties so growers can sustain incomes	Rooting ability; growth rate; suckering ability; shooting ability; and amenability to high-throughput multiplication	Seed entrepreneur and grower, based on ***how*** and ***where*** seed propagation is done

Breeding programs should collaborate closely with end-users to prioritize a minimum set of desired traits in new varieties. It is necessary to know which traits are deficient in the most grown varieties to determine which traits to improve, and those for maintenance at current threshold levels. Multi-disciplinarity will be essential and should thus consider insights of social and gender-scientists, economists, physiologists, food scientists, agronomists, molecular biologists, seed systems specialists and biometricians. "where" refers to the environments and/or geographical regions of the world where robusta is grown now or will be grown in the future. These options will determine trait priorities. "how" refers to the production systems used to grow robusta now and/or in the future, such as small-holder minimal input, technified monocropping under full sun, intercropping, and/or shaded agroforestry systems. These options will determine trait priorities. "who" in the context of a processor and/or roaster, refers to stakeholders that purchase, add value, and/or launch robusta products onto the market. These stakeholders will determine trait priorities, depending on their target market segments. "how" in the context of a processor and/or roaster refers to repeatable procedures adopted to produce robusta products destined for specific market segments. These processing procedures will determine trait priorities. "what" refers to a variety of products along the value chain (such as raw, unroasted, unprocessed, and/or semi-processed robusta), that are purchased for value addition. These products will determine trait priorities when purchasing. "how" and “where” in the context of a seed multiplier refers to the recommended seed production methods utilized to produce either seed-propagated or clonally-propagated robusta varieties. The recommended seed production method will determine trait priorities for seed propagation.

### 3.2 Strategies to drive genetic gains in *C. canephora*

Once target product profiles have been developed in consultation with key stakeholders, the next step is the design and optimization of breeding processes to deliver the new variety that meets stakeholder expectations. Here, we focus on efforts tailored towards improving breeding processes (i.e., efficiency in delivering new breakthrough varieties into farmers’ fields within reasonable timeframes), as this will make robusta breeding more impactful. Key components of breeding, such as strategic exploiting genetic variance, selection accuracy and intensity, and shortening of breeding cycles (Cobb et al., 2019; Yan, 2021), should be meticulously manipulated, all building on previous achievements (**Table 6**).

**Table 6:** Quantitative insights into operations, knowledge generated, and progress attained by selected robusta breeding programs.

Country	Traits and trial design	Quantitative insights	References
Indonesia	**Traits**: yield, and pest and disease resistance **Design**: single-tree and multiple-tree plots; evaluations done over 5 years	Change from mass to progeny-based selection led to >50% higher yield Yield gains attained through change from “single-clone” to “multiple-clone” deployment	Ferwerda (1948)
Ivory Coast	**Traits:** productivity; vigor; architecture; quality; drought; pest and disease resistance **Design**: single-tree plots; evaluations for 2–4 years	100 progenitors Overall performance of inter-group > intra-group hybrids Higher selection accuracy for bean size than for caffeine content	(Leroy et al., 1993)
Ivory Coast	**Traits:** vigor and yield **Design:** single-tree plots; evaluations for 5 years	Genetic gain for yield from BC1 to BC2 ~22% following *C. canephora* x *C. liberica* crosses	(Yapo et al., 2003)
Cameroon	**Traits**: bean weight; pea berry percentage; cherry yield **Design**: Single-tree plots with multiple stems/trees; evaluations done for 14 years	22 progenitors Yield had higher heritability (H^2^ = 0.43) than pea berry traits (H^2^ = 0.09) and berry dimension traits (H^2^ = 0.06 to 0.18)	Cilas and Bouharmont (2005)
Brazil	**Traits**: maturation; plant architecture; yield and disease resistance **Design**: Single-tree plots evaluating conilons (71); robusta (56) and their respective hybrids (20 families). Evaluations done over 5–7 years	Selection accuracy for conilons (0.53 to 0.89), robustas (0.53 to 0.87) and for hybrids (0.05 to 0.67) Highest correlations between yield and vigor (r=0.42; conilon and r=0.36 for robusta) while for hybrids it was yield and canopy diameter (r=0.44)	(Alkimim et al., 2021)
Brazil	**Traits:** 20 traits **Design:** 103 and 118 of intermediate and premature populations; 5 plants/plot with 3 replicates at 2 sites. Evaluations over 4 years	Broad-sense heritability (H²) ranged from 0.15 to 0.90 for morpho-agronomic, disease resistance, post-harvest, and yield traits. Finlay-Wilkinson regression has relatively higher prediction accuracy (−0.03 to 0.59) than AMMI (−0.12 to 0.39)	(Adunola et al., 2023)

Datasets from selected robusta breeding operations in the 1930s, 1990s, early 2000s, and in 2023. Progress achieved demonstrates interconnectivity of the adopted breeding steps; types of varieties developed using specific germplasm; human resources, and technology available. AMMI, Additive Main-effects and Multiplicative Interactions. Traits: This refers phenotypes (attributes) evaluated in the experimental trial. Design: This entails the experimental field layout, plot assignment, number of clones evaluated and/or duration of field evaluations.

Standing genetic variation provides the basis for any breeding program relying on genetic crosses to develop new varieties. Priority traits for robusta breeding should therefore exhibit appreciable genetic variation if they are to be improved. In addition, these traits should be heritable, as previously demonstrated when genetically distant robusta parents (i.e., parents assigned to different genetic groups or clusters based on empirical data) are crossed (Leroy et al., 1993; Eskes and Leroy, 2004; Alkimim et al., 2021). However, in case genetic variation for a desired trait is insufficient, with no clear variation, then systematic trait discovery, germplasm enhancement and pre-breeding could be explored as done previously through hybridisation campaigns between *C. canephora* and C. *liberica* (Yapo et al., 2003), and between *C. canephora* and C*. congensis* (Ouattara et al., 2021). Systematic hybridizations within genetic group can also be explored as done in Uganda between ‘Congolese’ x ‘Congolese’ during the search for coffee wild disease resistance (Musoli et al., 2013).

Gene banks and/or wild relatives at sites of origin are excellent sources for such traits and/or genetic diversity currently missing in breeding materials (Bramel et al., 2017; Davis et al., 2023, 2025; Ngure and Watanabe, 2024). Across the globe, breeding programs have incorporated a relatively wide range of parental lines for crosses, ranging from fewer than 30 to up to 100 progenitors (**Table 6**). The optimization of the number of parents, number of crosses, and progeny per cross depends on the depth of knowledge of parental breeding values, the use of optimal cross prediction and available resources.

In addition, selection accuracy, defined as the correlation between predicted and observed breeding values should be enhanced; higher selection accuracy results in faster genetic gains (Cobb et al., 2019). Selection accuracy and heritability are tightly interconnected, and trait heritability should be routinely assessed to guide breeding decisions. In practice, modest heritability values (i.e., ≤ 0.4) can attain reasonable accuracy (i.e., ≥ 0.4) as observed in maize empirical studies (Crossa et al., 2014).

Heritability estimates and correlations for selected robusta traits are highlighted in **Table 6**. Several empirical studies have also estimated trait heritability (Montagnon et al., 2003; Akpertey et al., 2020). These estimates should inform decisions on which traits to prioritize in regard to trial dimensions and stage of evaluation. Importantly, enhanced experimentation consistently leads to greater trait heritability, which drives higher genetic gains. Examples of such experimentation include the consideration of spatial variation in the field, adequately testing entries in the target environments, enforcing skilled field plot management, and using high-throughput phenotyping equipment and tools (Gezan et al., 2006; Cobb et al., 2019; Yan, 2021; Bassi et al., 2024).

The plot size of robusta experimental fields has varied markedly among breeding programs, from single-tree plots to multiple-tree plots with 5, 9, 10, 24, 49, 100 or up to 1,100 trees per plot (Ferwerda, 1948; Butters, 1964; Musoli et al., 2013; Mistro et al., 2019; Akpertey et al., 2020). Non-replicated trials with up to 3,500 genotypes were even performed (Santin et al., 2019). Notably, incomplete block designs, such as p-reps (partially replicated designs) or row-column layouts (Cullis et al., 2020; Piepho et al., 2021), have not been commonly used in most robusta breeding trials (**Table 6**), even though these designs attempt to strike a balance between selection accuracy, genetic gain, and operational cost per plot, and should thus be considered more often.

Selection intensity—that is, the proportion of the population selected as parents or those individuals advanced to the next evaluation stage—should also be the subject of optimization. To practically achieve higher selection intensity within the allotted budget, breeding programs should integrate genomic selection, as is currently being explored in Brazil (Ferrão et al., 2019; Adunola et al., 2023). Another key approach is implementing low-cost pre-screening of large populations with validated genetic markers or the application of high-throughput phenotyping tools (Cobb et al., 2019; Bassi et al., 2024).

Furthermore, sparse testing experimental designs (i.e., subset of genotypes evaluated in different sites, with a small set of clones evaluated at every site), although not routinely used for robusta field trials, can allow the evaluation of many individuals, particularly in early evaluation stages, thus enhancing selection intensity. Finally, a citizen science approach whereby farmers receive a set of individual entries (i.e., between 10 to 30 entries) for testing under their own conditions (De Sousa et al., 2024), offers further prospects for raising selection intensity, as several entries are evaluated by farmers (Bassi et al., 2024). The above strategies should help evaluate a reasonable number of individuals (for example up to 1000 single trees) in early seedling evaluation stages to achieve high selection intensity.

Another critical strategy to accelerate genetic gains is to shorten the duration of breeding cycles which will simultaneously lower costs (Cobb et al., 2019). Robusta has a long breeding cycle; the seed-to-seed generation following evaluation and selection, typically spans almost 20 years for new varieties to be released and/or for new proven progenitors to be crossed to generate the next cycle. Genomics-assisted selection should therefore be prioritized to reach shorter breeding cycles (Adunola et al., 2023, 2024; Rocha et al., 2025).

Breeding programs that rely solely on phenotypic selection should consider modifying or shortening the duration of field evaluations, for example by cutting back field evaluation durations from six to four years, based on trait correlations and predictions. Equally valuable is the adoption and use of selection indices that provide for simultaneous improvement of multiple traits, all aimed at maximizing the genetic merit of selections, building on previous experiences encountered in Brazil (Carvalho et al., 2019). These modifications may require breeding organizations to adopt a culture of continuous optimization and improvement (Cobb et al., 2019).

For instance, indirect selection for juvenile vegetative traits, such as the number of lateral branches and trunk cross-sectional area, has been suggested as an effective predictor for cherry yield at later stages during reproductive growth (Akpertey et al., 2019); selecting for these early traits could substantially shorten the duration of field testing. Furthermore, practical observations in Ghana indicate that averaging yield assessments over the first three years is as efficient as a seven-year average for overall cherry yield, providing another valuable method to accelerate field testing (Anim-Kwapong et al., 2011). Similarly, strong and positive correlations of 0.84 and 0.82 were reported between the third and fourth cherry harvests, respectively, and average bean production in Brazil (Ferrão et al., 2019), supporting the notion that field testing duration can be shortened.

## 4 Conclusions and future perspectives

Fluctuating coffee prices, the effects of climate change on green bean supplies, and the multi-faceted challenges of smallholder farmers all point to the need for greater innovation and global partnerships in coffee production and breeding, to maintain the profitability and sustainability of this crop species. While robusta consumption continues to grow across multiple market segments, the same is not universally true for on-farm productivity. As the top four agricultural value chains (cereals and grains; horticulture; livestock and dairy; and roots and tubers) have taught us, farmer productivity can be dramatically improved through access to high-performing varieties augmented by appropriate agronomic, financial, and social support.

Plant breeding is a long-term endeavor requiring investment and well-coordinated global opportunities. Indeed, the Consultative Group system (https://www.cgiar.org/) and the Gates Foundation have made significant strides in upgrading global plant breeding as coordinated by the excellence in breeding platform serving multiple crops (Gorjanc et al., 2021). However, coffee has not been part of such global initiatives until very recently with the launch of Innovea (https://worldcoffeeresearch.org/programs/global-breeding-network), a global arabica coffee breeding network led by World Coffee Research. This arabica breeding network is focused on population improvement and has been designed with a pre-competitive model that provides improved germplasm to participating countries for further development into finished varieties.

In a pre-competitive paradigm, many partners or institutions work together to conduct foundational research to solve common challenges that benefit the entire industry before they begin to compete. The development of shared tools, data systems, and breeding strategies that benefit both public and private breeding programs (Gorjanc et al., 2021) without direct market competition is a classic example of pre-competitive model under the Consultative Group.

A robusta breeding network, modelled after the Innovea arabica network, is currently in development with a goal of addressing some of the biggest challenges facing many robusta breeding programs operating today. The design of the network aims to include harmonized trait screening, product selection and/or field testing within frameworks of multi-environment trials to allow cross-comparison of data. This robusta breeding network will also facilitate the legal exchange of useful germplasm and the development and use of validated tools, techniques, and methods such as genomic selection, marker-assisted selection, and high-throughput phenotyping to enhance capacity across all breeding programs.

**Figure 1:**
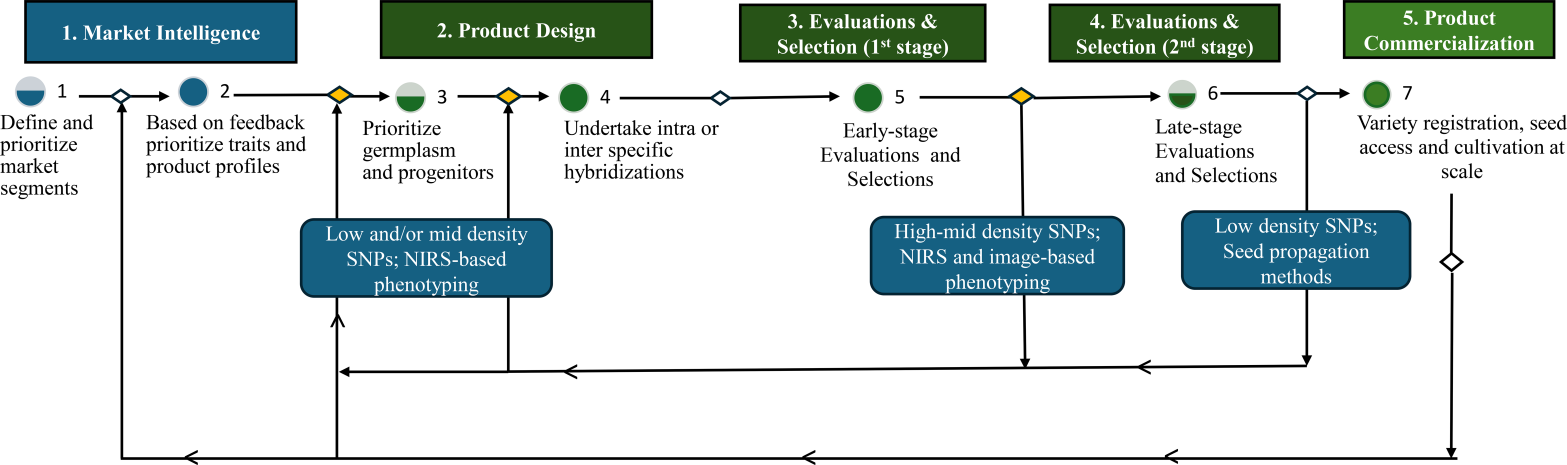
Main breeding stages and processes that integrate modern breeding tools and provide for feedback loops. We highlight three entry points for modern breeding tool integration. First, operationalizing processes 3 and 4, that entail strategic progenitor selection and crossing; this process could benefit from low and/or medium SNP density markers to maximize progeny performance. Proven phenotyping tools (e.g., for cup quality assessment) can be integrated. Second, operationalizing process 5 that entails evaluation of several individuals at single and/or multiple sites for several years; operational costs and timelines associated with these evaluations can be substantially reduced through use of genomics-assisted selection; proven instrument-based phenotyping tools can be integrated to reduce drudgery while increasing efficiency. Third, operationalizing processes 7, that reinforces genetic conformity prior to seed propagation and/or commercial production; this process can benefit from low density SNP markers. Feedback from key stakeholders helps determine new trait priorities, modify product profiles and to prioritize progenitors for hybridization. Collectively, these interventions will make robusta breeding outcomes more inclusive, impactful and durable.

Clearly, the current and future challenges faced by farmers and industry will necessitate strategic integration of modern breeding tools to complement conventional breeding to make it more responsive to climate change and evolving market needs. Proven and modern breeding tools such as marker-assisted selection, genomic selection, speed breeding, instrument-based phenotyping (like near-infrared spectroscopy and artificial intelligence-driven phenotyping) and seed propagation at scale, are prioritized wherever drudgery, accuracy and complexity are obstacles. Accordingly, we highlight three entry points for maximizing the impact of modern tool integration (**Figure 1**).

By understanding and addressing their challenges together, robusta breeding programs have an opportunity to coordinate efforts and deliver local, lasting impact in their respective countries, marking the start of addressing the supply and sustainability goals of the global coffee industry. Pre-competitive international collaborations among industry, academia, and governments is a powerful approach that focuses investment on key priorities and accelerates the development of innovations that farmers need.
